# Supplementary material for: Fully implanted battery-free high power platform for chronic spinal and muscular functional electrical stimulation
Source: Nat Commun. 2023 Nov 30;14:7887. doi: 10.1038/s41467-023-43669-2 (PMC10689769; doi:10.1038/s41467-023-43669-2)
Supplement: Supplementary file 1 — Supplementary Information [file 41467_2023_43669_MOESM1_ESM.pdf]

Fully Implanted Battery-Free High Power Platform for Chronic Spinal and Muscular Functional Electrical Stimulation

Alex Burton[1†], Zhong Wang[2†], Dan Song[3], Sam Tran[2], Jessica Hanna[1], Dhrubo Ahmad[1], Jakob Bakall[1], David Clausen[1], Jerry Anderson[1], Roberto Peralta[1], Kirtana Sandepudi[3], Alex Benedetto[4], Ethan Yang[2], Diya Basrai[2], Lee E. Miller[2,3,4,5], Mathew C Tresch[3,5,6], Philipp Gutruf[1,7,8]

[1]Department of Biomedical Engineering, University of Arizona; Tucson, AZ 85721, USA.

[2]Department of Neuroscience, Northwestern University; Chicago, IL 60611, USA.

[3]Department of Biomedical Engineering, Northwestern University; Evanston, IL 60208, USA.

[4]Interdepartmental Neuroscience, Northwestern University; Chicago, IL 60611, USA.

[5]Department of Physical Medicine and Rehabilitation, Northwestern University; Chicago, IL 60611, USA.

[6]Shirley Ryan AbilityLab; Chicago, IL 60611, USA.

[7]Bio5 Institute and Department of Neurology, University of Arizona; Tucson, AZ, 85721, USA.

[8]Department of Electrical and Computer Engineering, University of Arizona; Tucson, AZ 85721, USA.

Correspondence should be addressed to MCT (Email: m-tresch@northwestern.edu) and PG (Email: pgutruf@email.arizona.edu).

[†]These authors contributed equally to this work

a

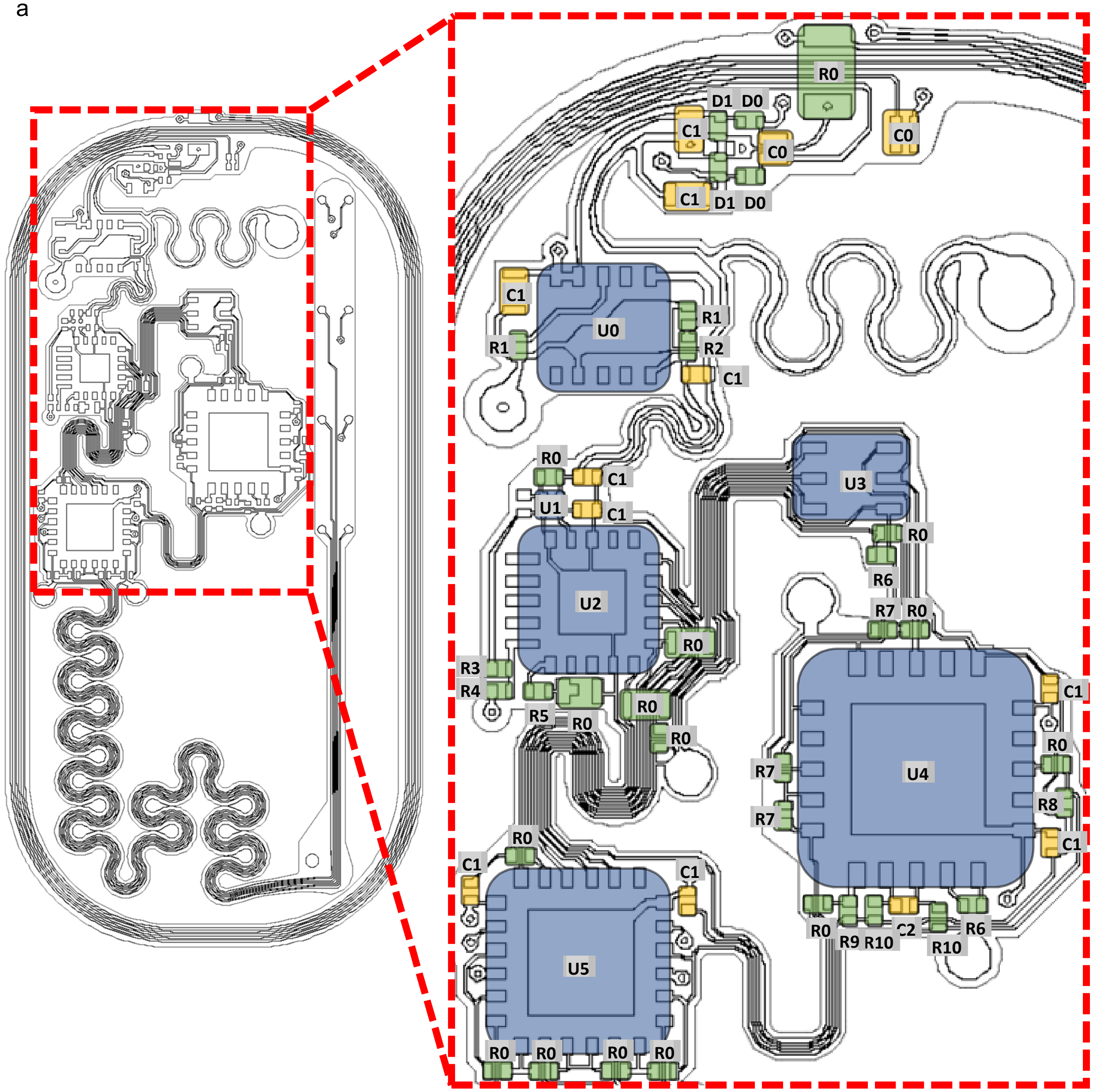

Supplementary Fig. 1. Schematic view of the circuit and component placement.

a

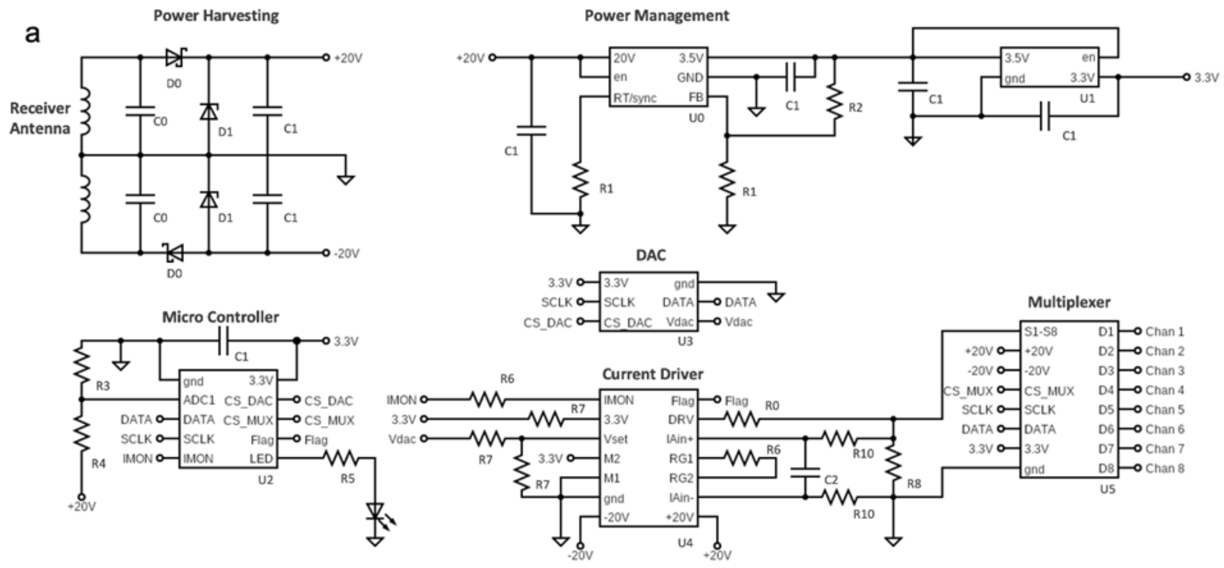

b

R0	0 Ω
R1	150 kΩ
R2	47 kΩ
R3	4.7 kΩ
R4	300 Ω
R5	150 kΩ

R6	10 kΩ
R7	20 kΩ
R8	1 MΩ
R9	1 kΩ
R10	2 kΩ
C0	44 pF

C1	2.2 μF
C2	200 pF
U0	MAXM17532 (Step Down)
U1	NCP3335ADM330 (LDO)
U2	Attiny 84A (MCU)
U3	DAC5311 (DAC)

U4	XTR300 (Current Mirror)
U5	ADGS5414 (MUX)

Supplementary Fig. 2. Circuit Diagram of the implantable device. (a) Schematic. **(b)** List of the main electrical components on device.

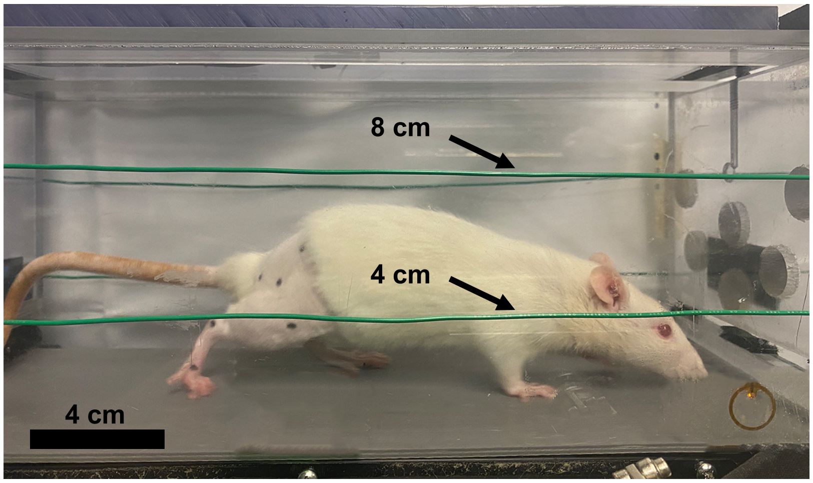

Supplementary Fig. 3. Photograph of rat showing relative position of the implanted device and cage height.

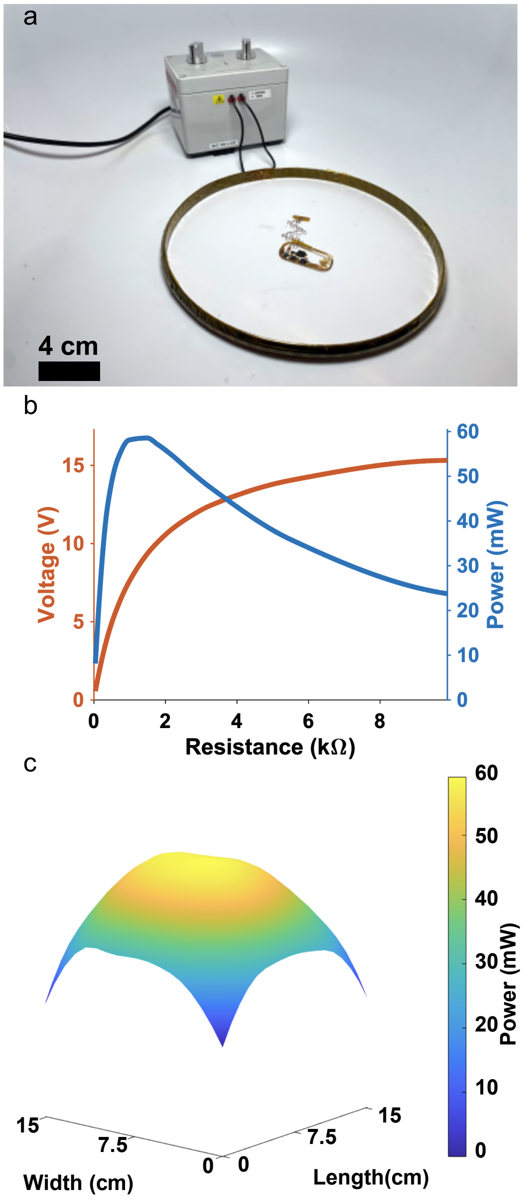

Supplementary Fig. 4. Electrical characterization of the circular antenna. (a) Photograph image of the circular antenna design. **(b)** Voltage power curve in the center of the circular antenna design at 3 W of RF power. **(c)** Spatial power harvesting capability of the circular antenna with a 2 kΩ load to match the equivalent load of the circuit at 3 W of RF power.

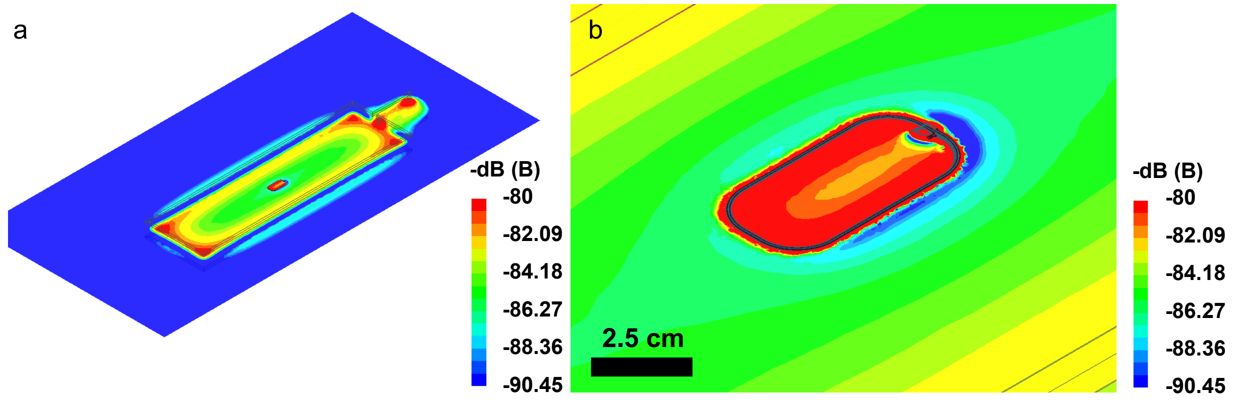

Supplementary Fig. 5. Simulation of the B-field generated by the power casting system. (a) Zoomed out view of the B-field showing the cage and device in the center of the cage. **(b)** Zoomed in view of the B-field around the device antenna.

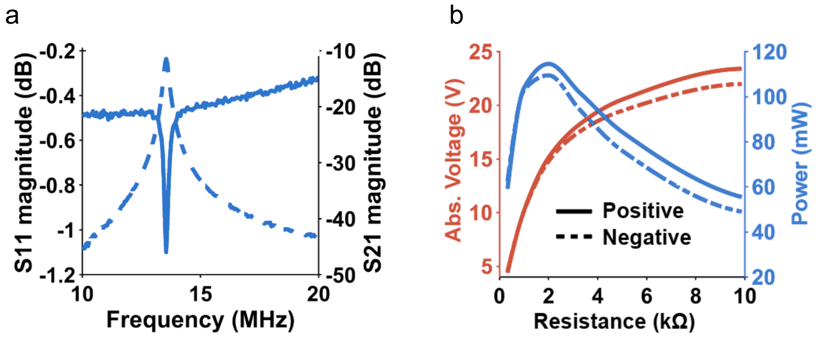

Supplementary Fig. 6. Power harvesting of resonant antenna design. (a) S11 and S21 magnitude of the resonant antenna design in the center of a two turn 56 cm x 16 cm cage with a spacing of 4 cm between turns. **(b)** Voltage power in the center of a 56 cm x 16 cm cage showing both negative and positive voltage supplies.

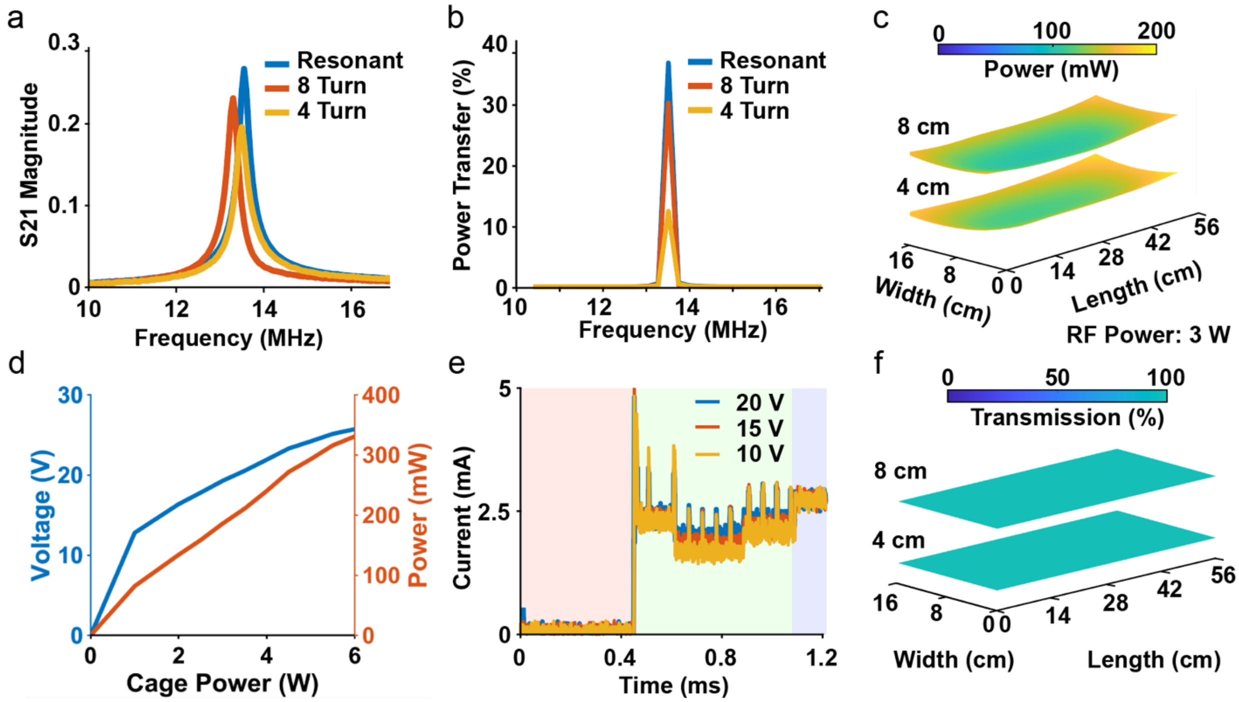

Supplementary Fig. 7. Characterization of wireless power transfer and communication. (a) Measured magnitude of transmission coefficient |S21| between 3-antenna WPT containing a resonant coil and a conventional 2-antenna WPT with 4 and 8-turns respectively. **(b)** Simulated power transfer efficiency between 3-coil WPT containing a resonant coil and the conventional 2-coil WPT with 4 and 8-turns respectively. **(c)** Spatially resolved power harvesting capability at two heights in a 56 cm x 16 cm treadmill cage. **(d)** Power harvesting capability for the range of 2 W to 6 W of RF transmitter antenna power. **(e)** Power consumption of the device during start up with decreasing voltage compliance. **(f)** Spatial stability of the IR communication in a 56 cm x 16 cm cage at 3 W of RF power.

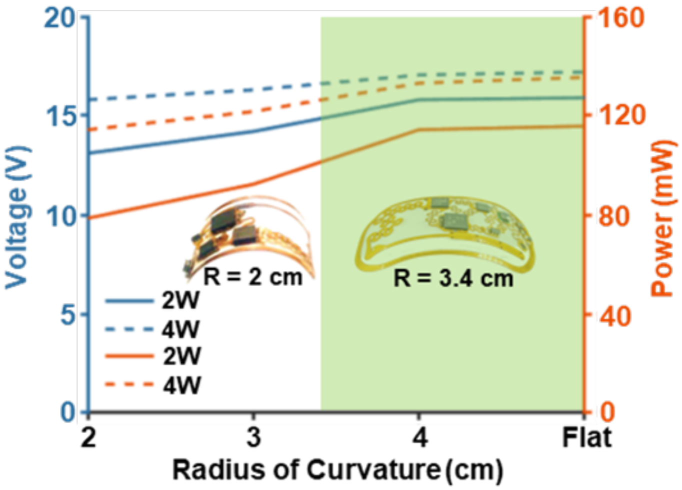

Supplementary Fig. 8. Bending characterization of the antenna measuring harvested power with a 2KΩ load for increasing radius of curvatures for 2W and 4W of RF power. Physiologically relevant range marked in green.

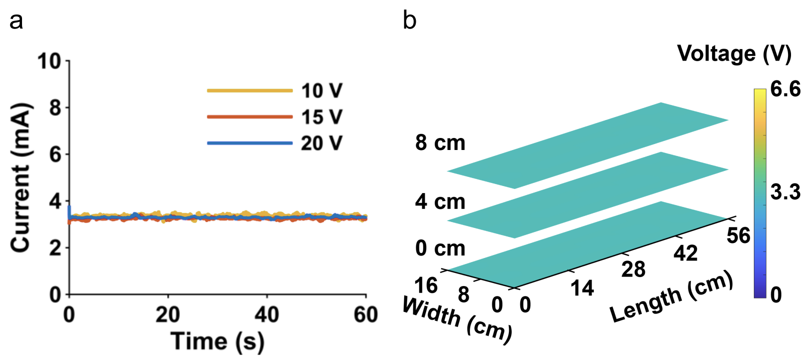

Supplementary Fig. 9. Step down characteristics. (a) Current consumption during stimulation of 1 mA with a 10 kΩ load across the stimulation electrode. **(b)** Digital voltage supply after stepdown and low voltage drop out regulator.

a

Component	XTR300	MUX	MCU	DAC	Step Down	Zener
Power (mW)	19.44	0.84	1.98	0.33	3.60	8.00

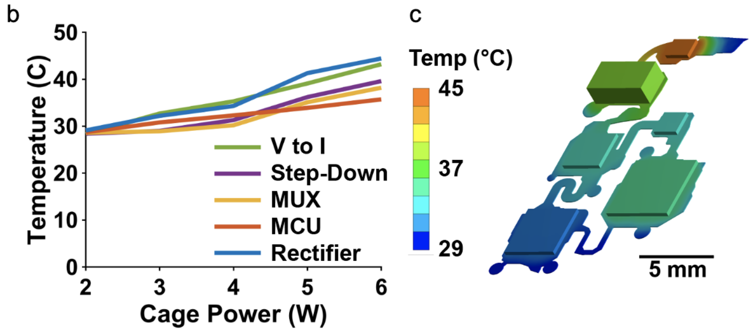

Supplementary Fig. 10. Thermal properties of the device during operation. (a) List of estimated power consumption of each active IC based on data sheet and operation time. **(b)** Direct thermal measurement of active components in increasing cage powers in air. **(c)** Finite element analysis of heat transfer on device surface in air with an RF power of 6 W.

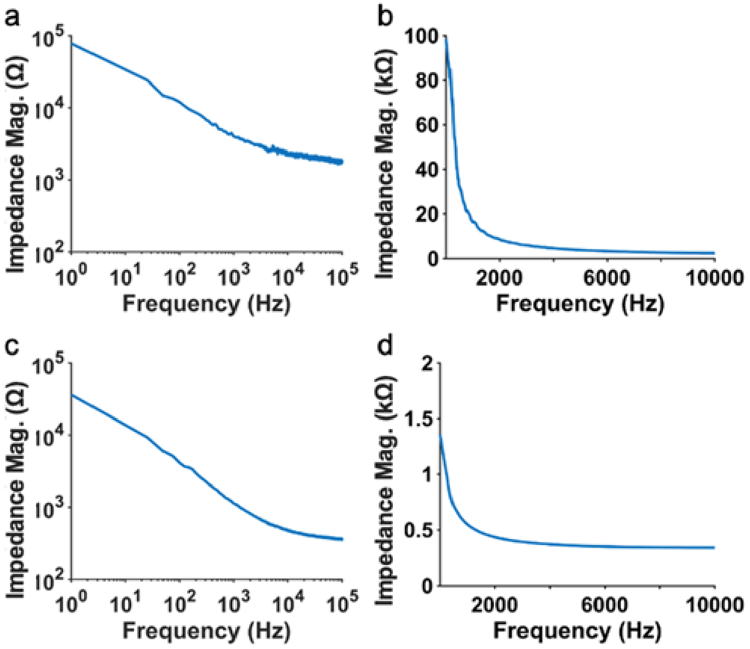

Supplementary Fig. 11. Electrode impedance magnitude in reference to the return electrode. **(a-b)** Impedance magnitude of the spinal electrode in a x1 saline solution in both log and linear graph. **(c-d)** Impedance magnitude of the stainless-steel muscle electrode x1 saline solution in both log and linear graph.

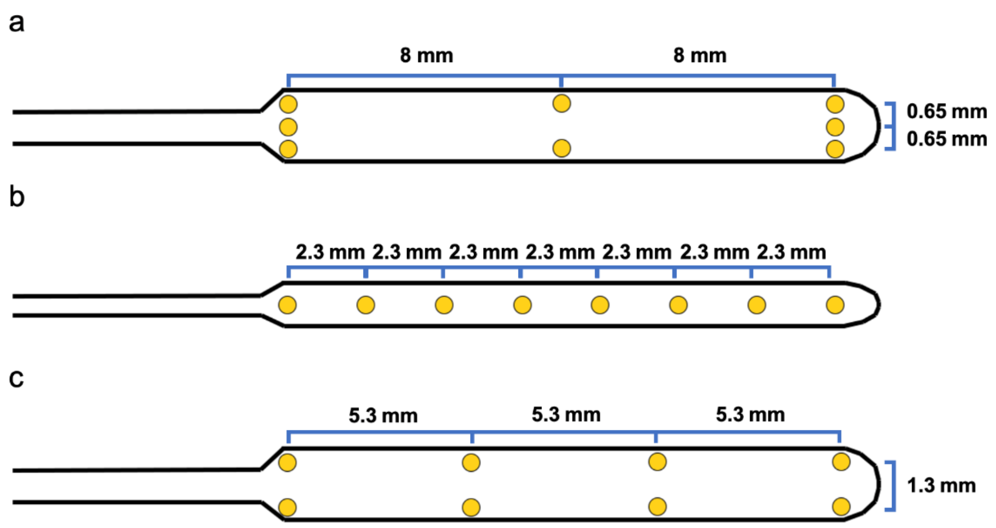

Supplementary Fig. 12. Electrode configurations drawings. (a) Array 1: 8 by 1 electrode array. **(b)** Array 2: 4 by 2 electrode array. **(c)** Array 3: Alternating 3 by 2 by 3 configuration.

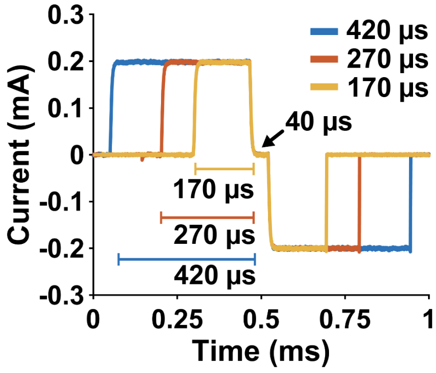

Supplementary Fig. 13. Wirelessly programmable control of biphasic stimulation pulse width duration with a fixed 40 µs interphase gap between anodic and cathodic phase.

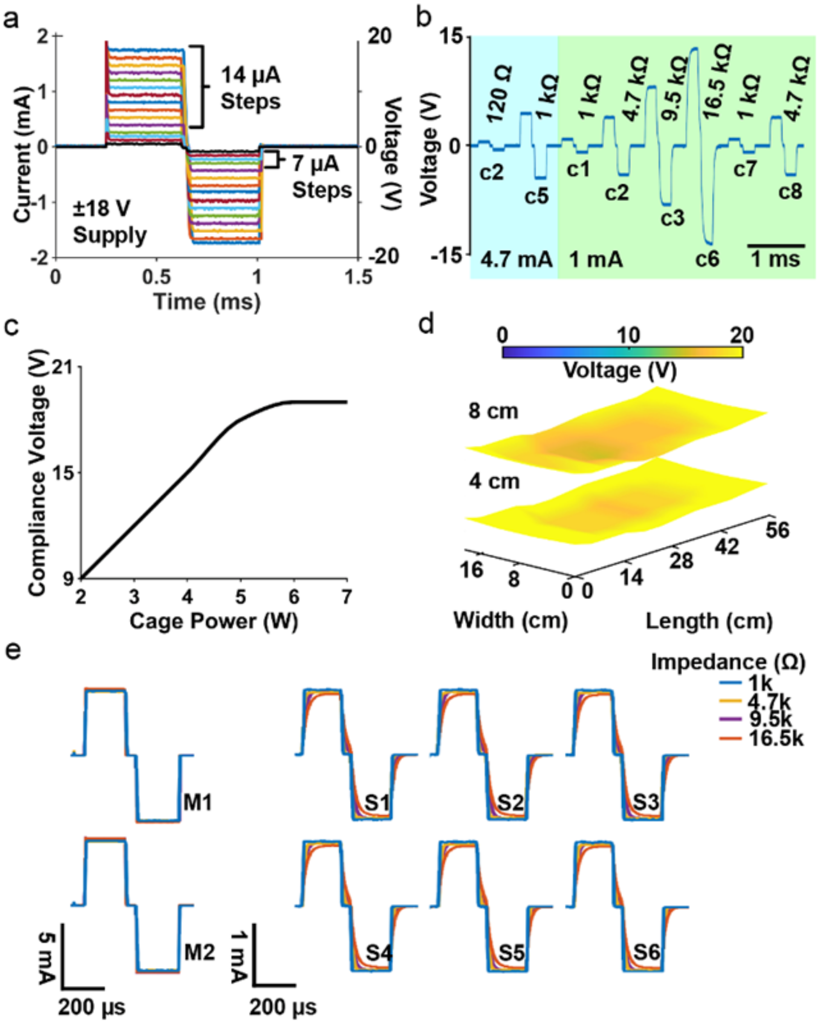

Supplementary Fig. 14. Current controlled biphasic stimulation. (a) Programmable stimulation amplitude for a maximum biphasic current of 1.8 mA. **(b)** Individualized current control over all stimulation channels. **(c)** Voltage compliance with respect to cage power while stimulating at 4.7 mA in saline. **(d)** Voltage compliance within the treadmill cage arena (56 cm x 16 cm) with a 5 W RF power. **(e)** Recorded current through various electrical loads from 1 kΩ to 16.5 kΩ for muscle electrodes labeled M1 and M2 and spinal electrodes labeled S1 through S6.

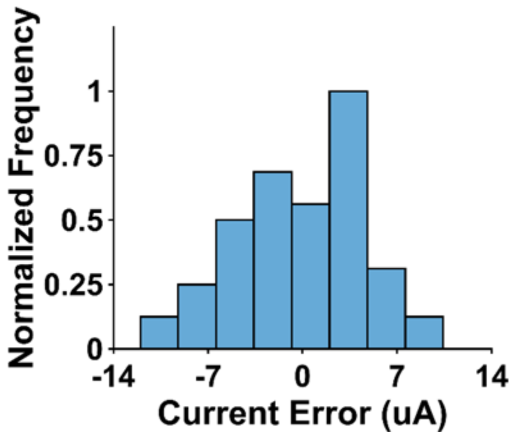

Supplementary Fig. 15. Measured current error over multiple stimulation cycles with a 10 kΩ load.

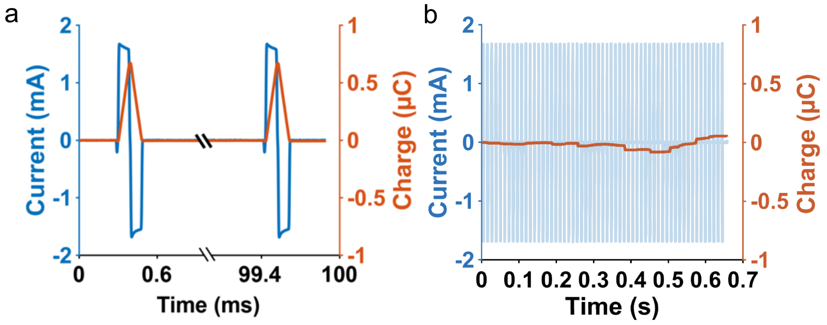

Supplementary Fig. 16. Stimulation charge. (a) Charge build up on the spinal electrode. **(b)** Net charge buildup on spinal electrode during current controlled biphasic stimulation.

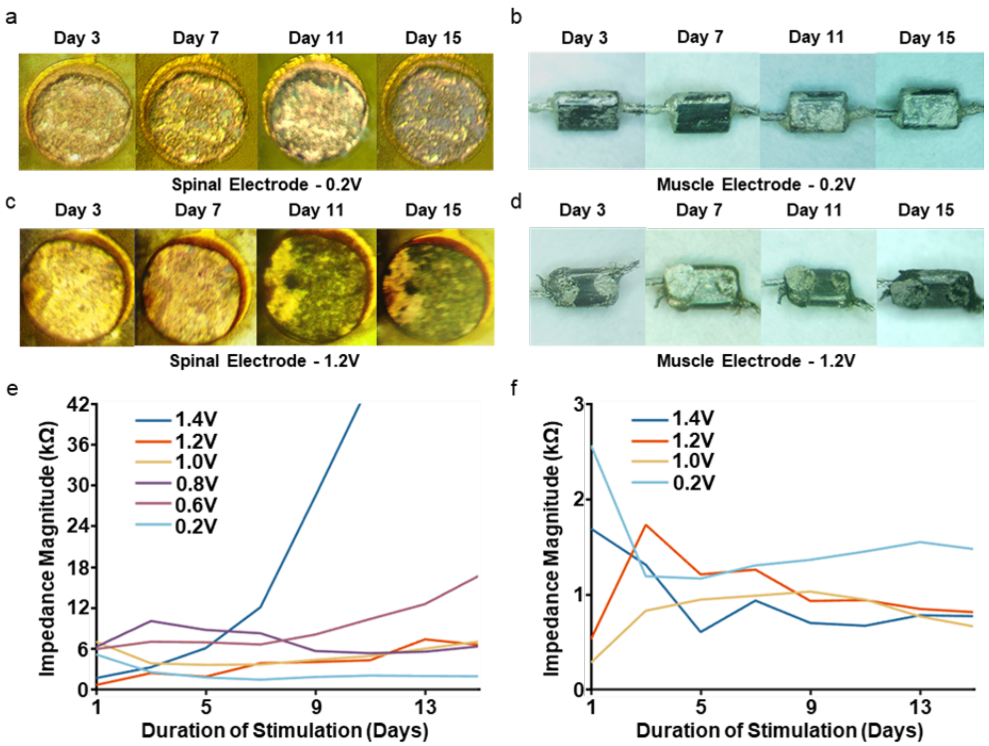

Supplementary Fig. 17. **Electrode degradation and changes for chronic accelerated electrode lifetime tests. (a)** Spinal electrode degradation after 3 days, 7 days, 11 days and 15 days of biphasic stimulation at ± 0.2 V at 1 kHz and 100% duty cycle **(b)** Muscle electrode degradation after 3 days, 7 days, 11 days and 15 days of biphasic stimulation at ± 0.2 V at 1 kHz and 100% duty cycle **(c)** Spinal electrode degradation after 3 days, 7 days, 11 days and 15 days of biphasic stimulation at ± 1.2 V at 1kHz and 100% duty cycle. **(d)** Muscle electrode degradation after 3 days, 7 days, 11 days and 15 days of stimulation at ± 1.2 V at 1 kHz and 100% duty cycle **(e)** Impedance changes over time for spinal electrodes at voltage within the water window and above. **(f)** Impedance changes over time for muscle electrodes within water window and above.

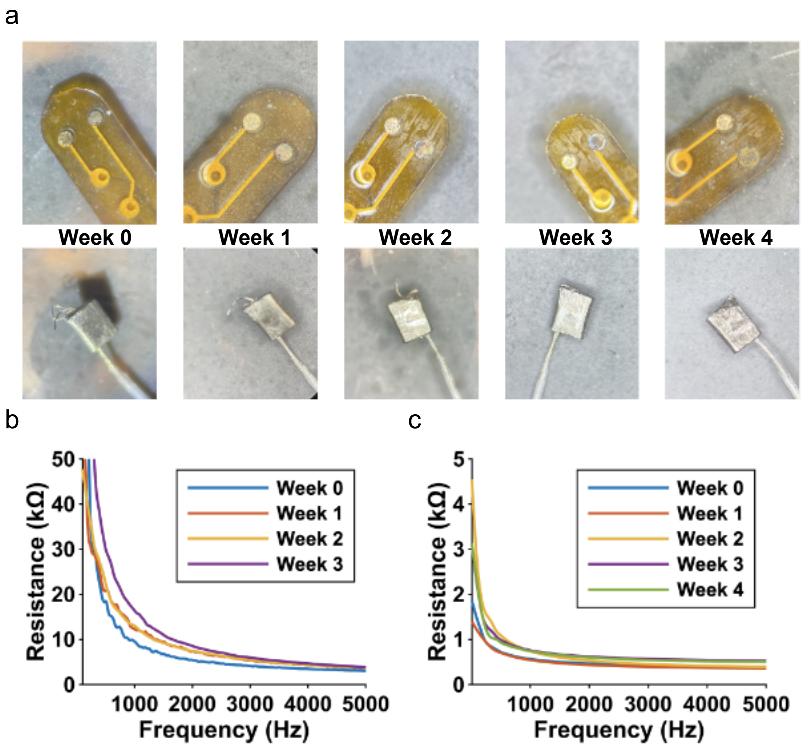

Supplementary Fig. 18. Continuous 1 mA stimulation in PBS. (a) Photograph image of the spinal electrode and muscle electrode stimulating in saline over four weeks. **(b)** Impedance sweep of the spinal electrode over multiple weeks. **(c)** Impedance sweep of the muscle electrode over multiple weeks.

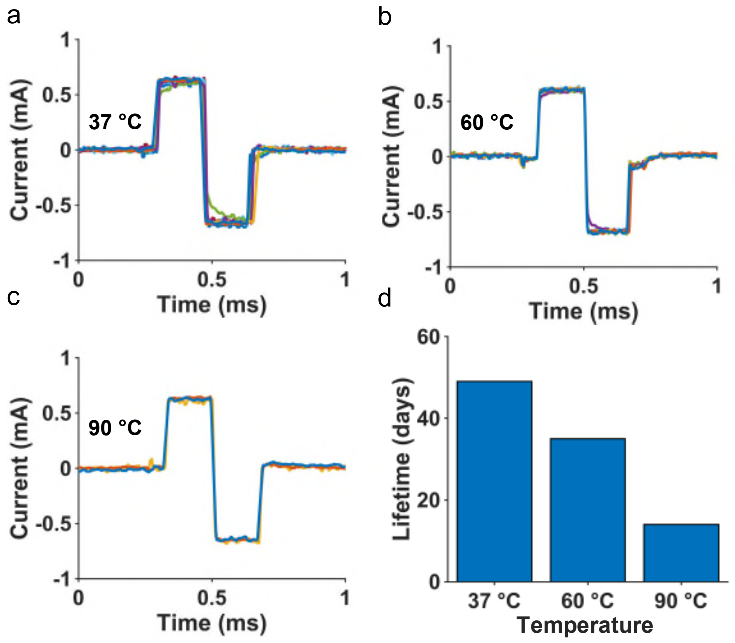

Supplementary Fig. 19. Accelerated rate test of devices in PBS. (a) Measured current at one-week intervals of a functional device in 37 °C. **(b)** Measured current at one-week intervals of a functional device in 60 °C. **(c)** Measured current at one-week intervals of a functional device in 90 °C. **(d)** Bar graph of the devices under accelerated rate tests at three thermal stresses.

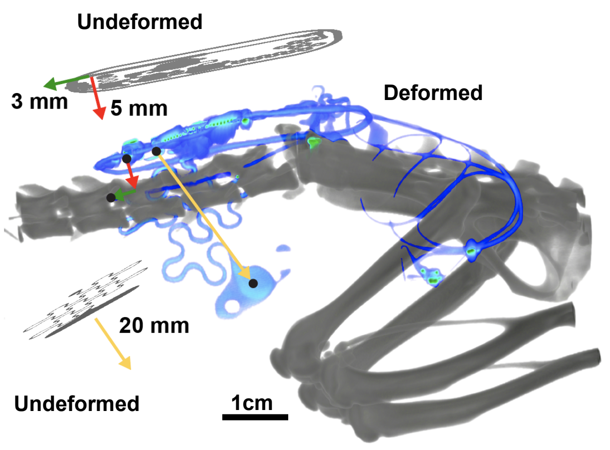

Supplementary Fig. 20. X-ray images of implanted device in a rat with arrows corresponding to direction of electrode displacement in the x and z axis.

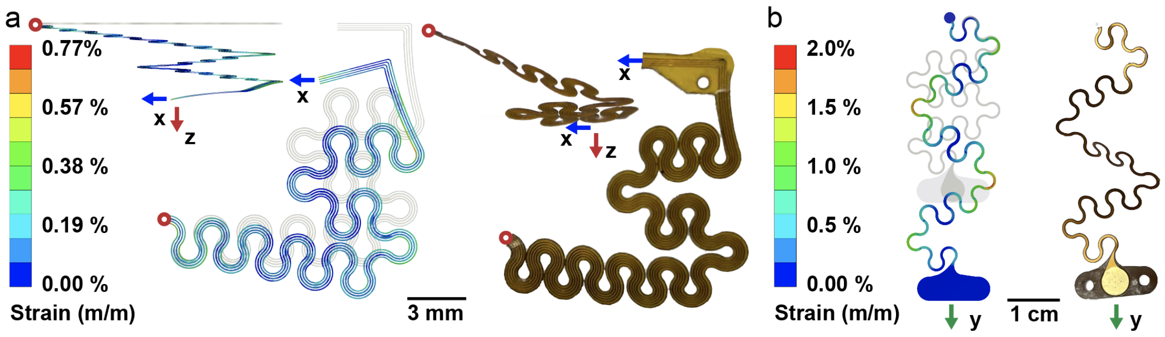

Supplementary Fig. 21. Mechanical simulations. (a) Simulation of direct strain on the copper layers as the spinal electrode is stretched matching deformations captured in CT images (left) and a photograph of the same deformation (right). **(b)** Simulation of direct strain on the copper layers as the return electrode is stretched matching deformations captured in CT images (left) and a photograph of the same deformation (right).

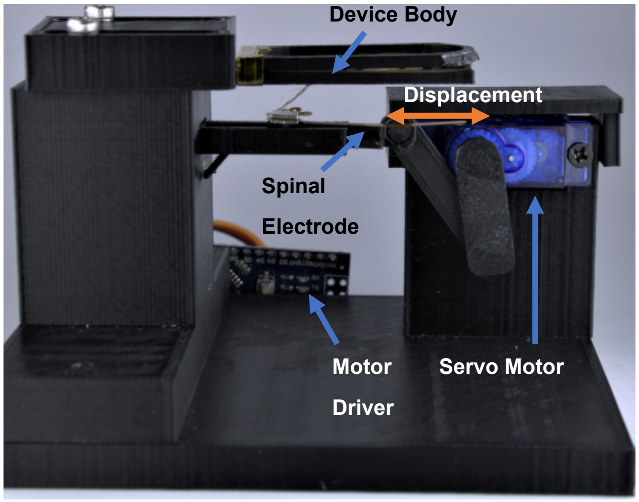

Supplementary Fig. 22. Mechanical stretching stage used to linearly deform the electrode over 1 million times in a configuration with fixed vertical offset to match implanted electrodes.

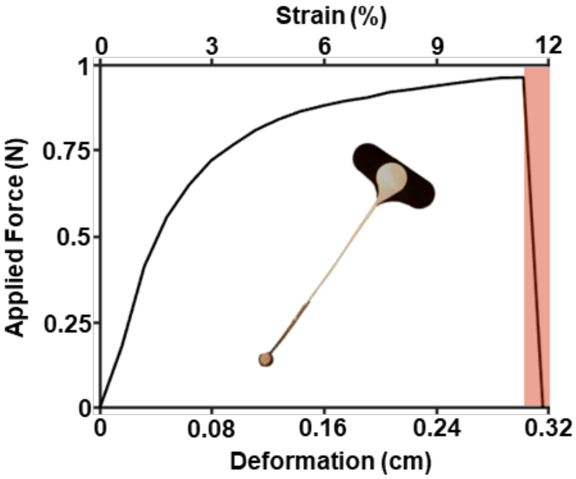

Supplementary Fig. 23. Strain stress curve demonstrating the mechanical properties of a linear interconnect.

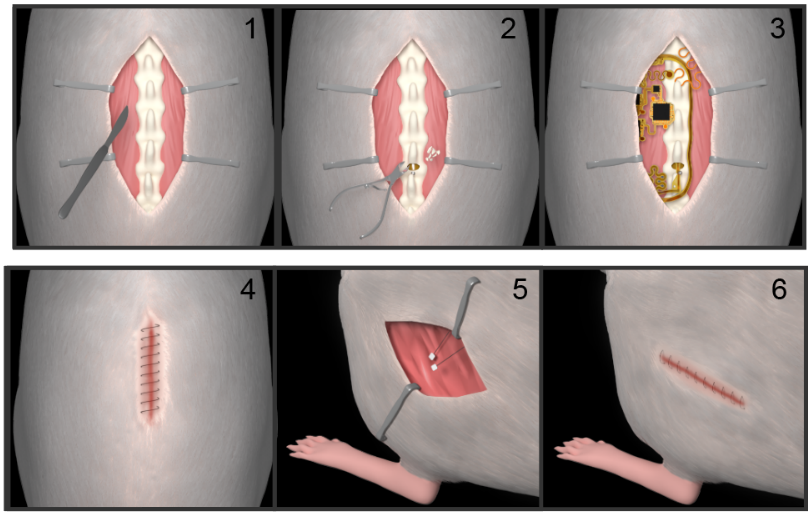

Supplementary Fig. 24. Rendered device implantation procedures (spinal implantation:1 to 4; muscle: 5 and 6)

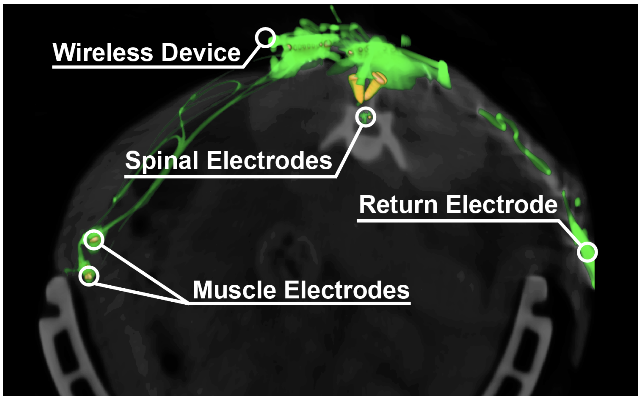

Supplementary Fig. 25. μCT reconstruction of the implanted device (cross-sectional view).

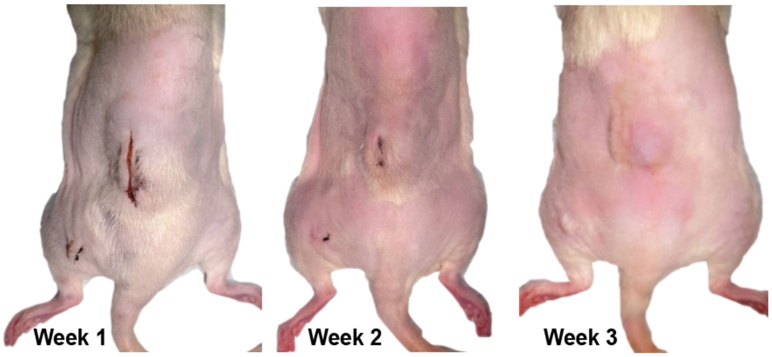

Supplementary Fig. 26. Incision healing at three days, one week, and three weeks post implantation.

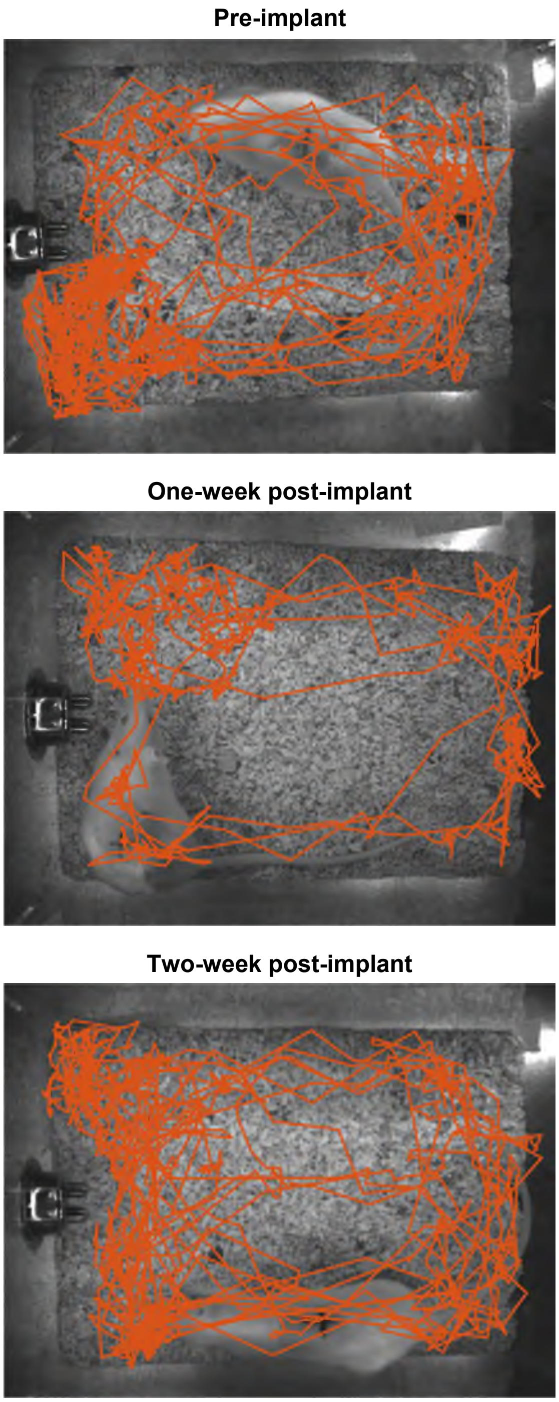

Supplementary Fig. 27. Animal in-cage trajectory from one week before implant, one week and two weeks after implant. Activity traces were collected over a 30 minutes period after animals were placed in the novel cage.

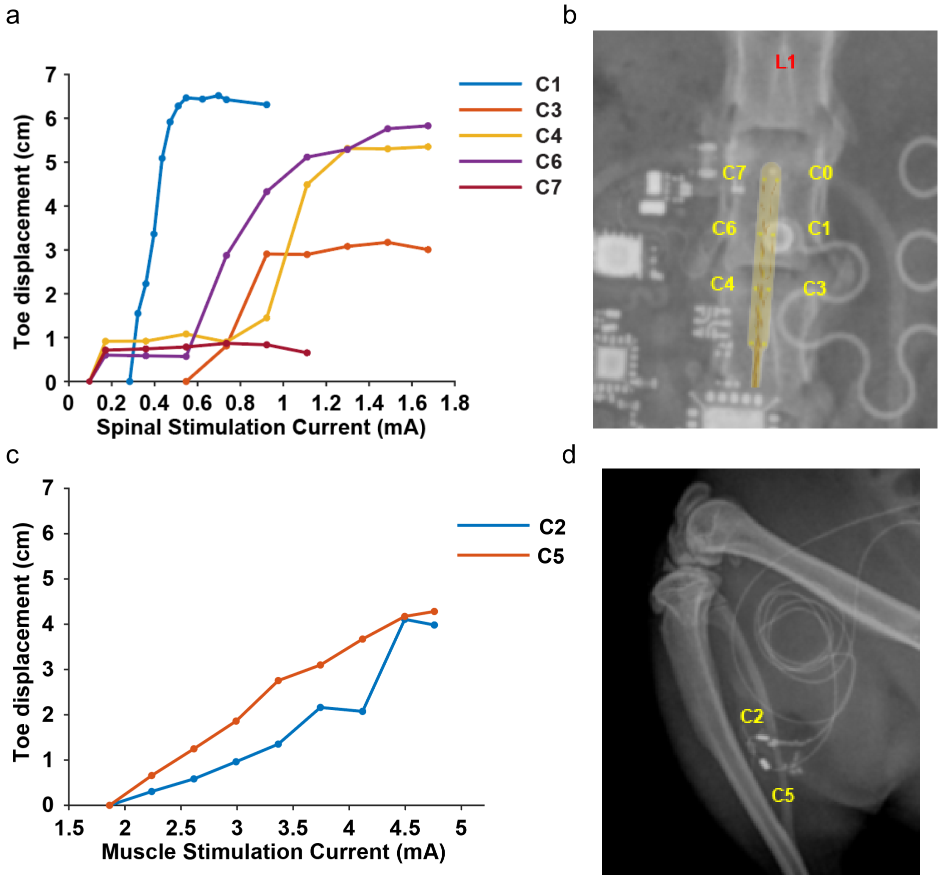

Supplementary Fig. 28. The recruitment curves of all channels from the same device indicated in Fig. 6f. **(a)** Recruitment curves from all spinal channels of the device. **(b)** Electrode position reconstructed based on fiduciary markers in x-ray and all the spinal channels are labelled. Note that stimulation of electrode c0 produced no responses and so is excluded from the figure. The recruitment curve for c1 is the same as illustrated in Fig. 6f. **(c)** Recruitment curves from all muscle channels of one device. **(d)** X-ray shows the muscle electrode position in the hindlimb. The recruitment curve for c5 is the same as illustrated in Fig. 6f.

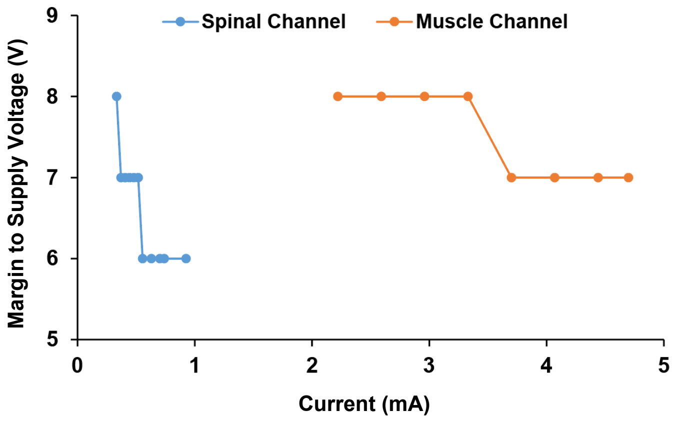

Supplementary Fig. 29. Margin to supply voltage (voltage difference between harvested voltage and the voltage needed to drive current at the stimulation site) during the stimulation trials illustrated in Fig. 6f. The information is communicated from the device through the IR interface and indicates how much voltage capacity remains after creating the specified current. Those experiments were performed with 3 W of RF power under the circular antenna setup (Fig. 6a).

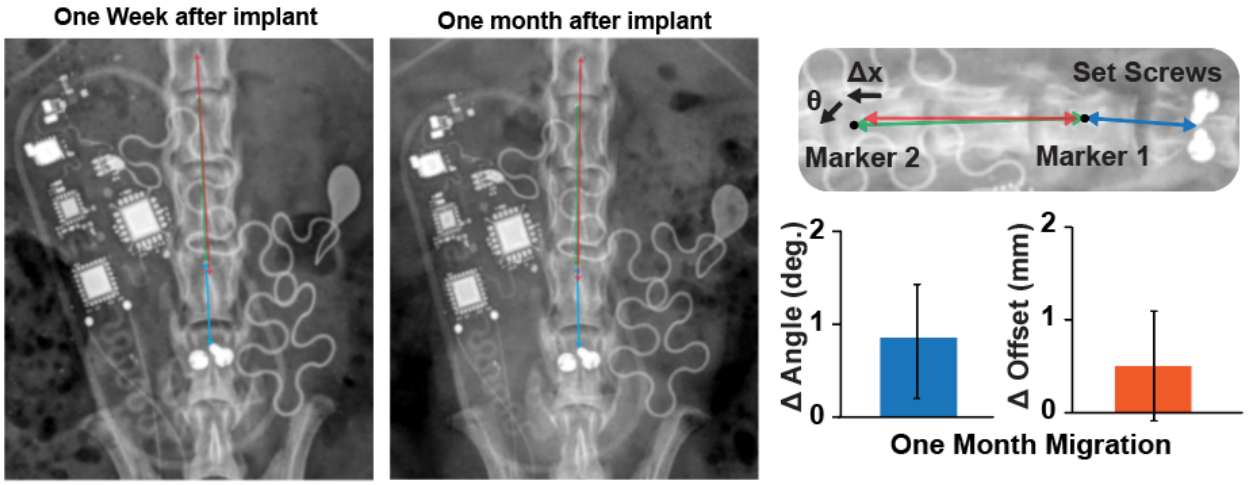

Supplementary Fig. 30. Left: X-ray comparisons of the implanted device of the same animal between one week and one month after the surgery. Both the epidural electrode and the subcutaneous device circuit almost stay where they were one month ago. The distances between the two fiduciary markers are measured as 18 mm in both images. **Right:** Upper: Identification of electrode panel axis (line between fiducial markers, in green), vertebrae axis (line between spinous processes, in red) and rostrocaudal location (distance between implanted screw and caudal fiducial marker, in blue). Lower: one-month changes of intersection angles between electrode and vertebrae axis (blue bar) and rostrocaudal location (orange bar) averaged from four implanted animals.

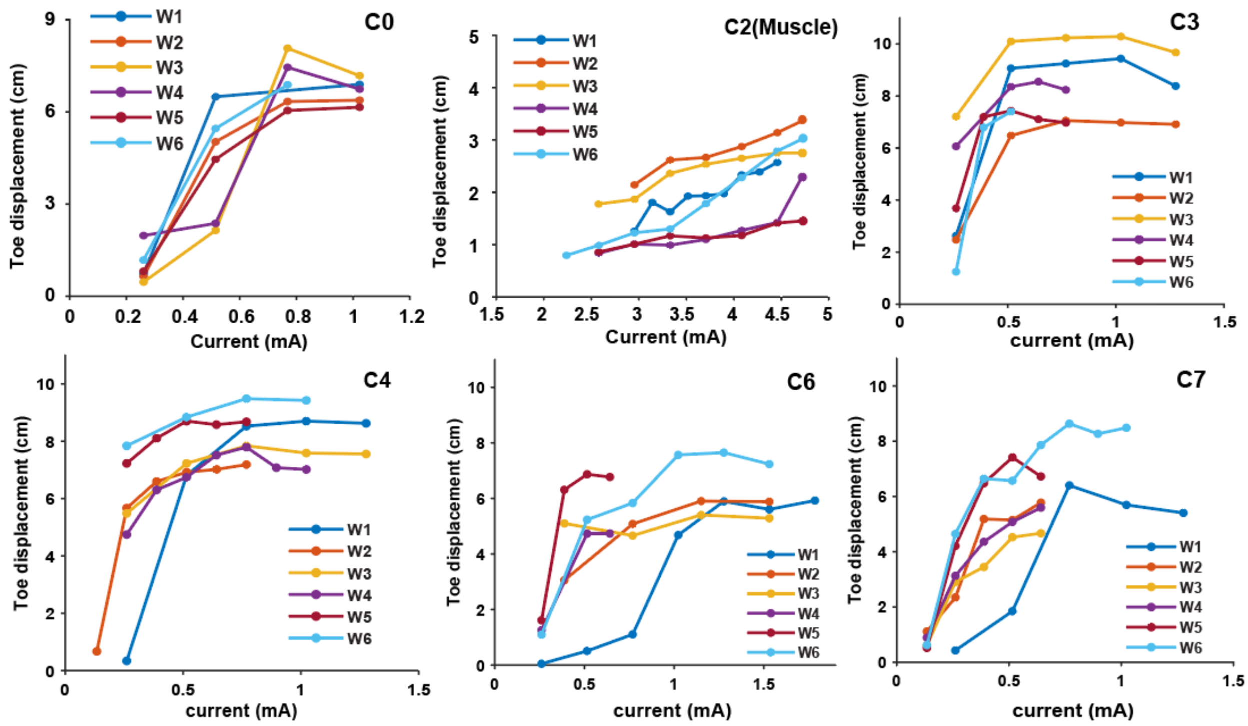

Supplementary Fig. 31. Chronic stability of all the channels (c0, c2, c3, c4, c6, c7) from the same device shown in Fig. 7a. Note that one spinal channel and one muscle channel didn't generate chronic data in this case. The recruitment curves stop when the evoked responses plateaued, voltage incompliance was indicated from IR, or intensities reached 1.5 mA safety threshold.

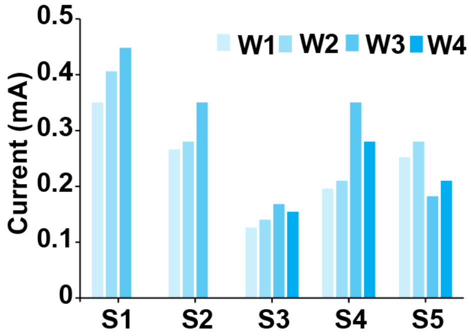

Supplementary Fig. 32. Comparison of recruitment thresholds for five spinal channels across four weeks.

No Stimulation

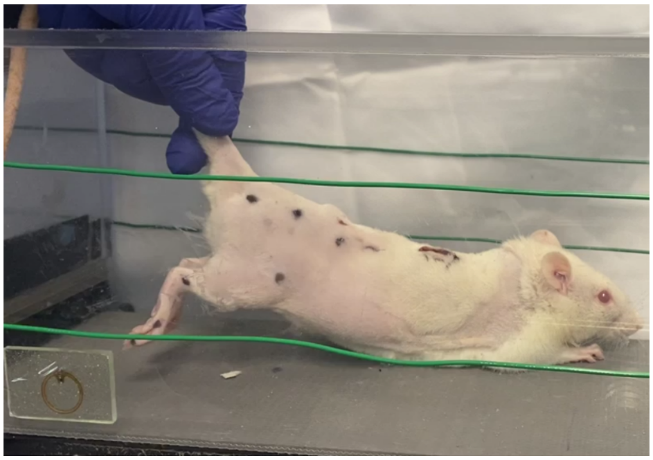

Stimulation

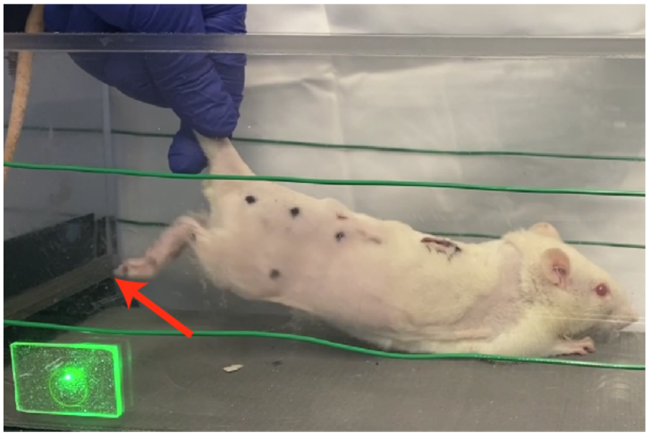

Supplementary Fig. 33. Photograph image of no stimulation and stimulation in an awake SCI animal. The experiment was conducted in a treadmill cage (56 cm x 16 cm) with two-turns antenna attached to the cage wall (4 cm apart, the same setup described in Fig. 1c). Stimulation of 1 mA was delivered at a frequency of 50 Hz and a duration of 100 ms on channel c3.

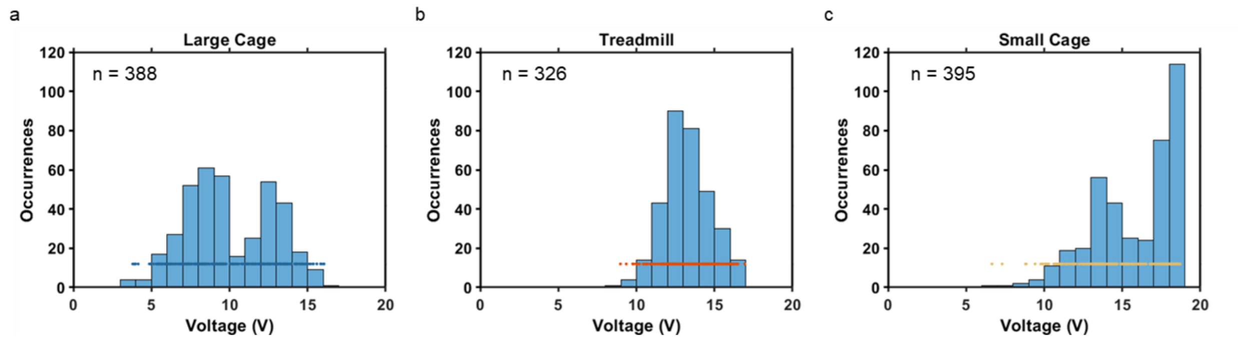

Supplementary Fig. 34. Histograms of voltage compliance distributions with overlaid dot plots in three enclosures of increasing size. (a) Large cage (29 cm × 36 cm). **(b)** Treadmill (16 cm × 56 cm). **(c)** Small cage (18 cm × 36 cm).

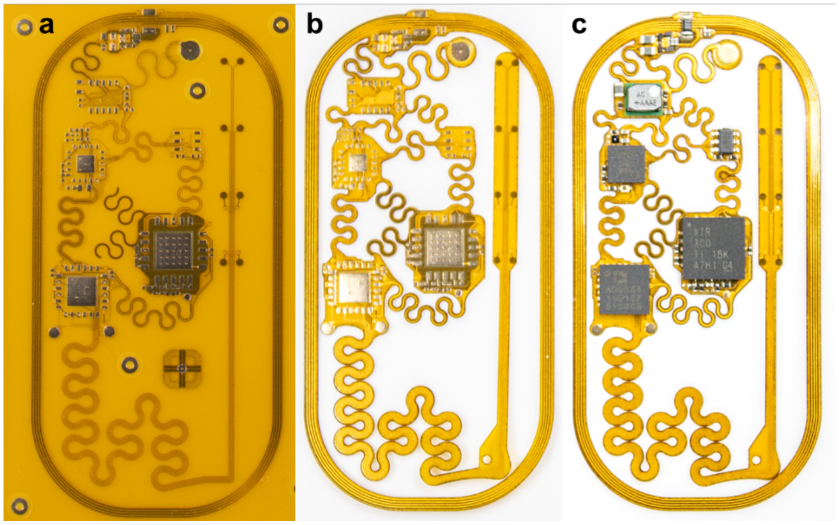

Supplementary Fig. 35. Implant panel top view. (a) Implant in panelized form with alignment markers **(b)** Implant depanelized with laser ablation process. **(c)** Device assembled with SMD components.

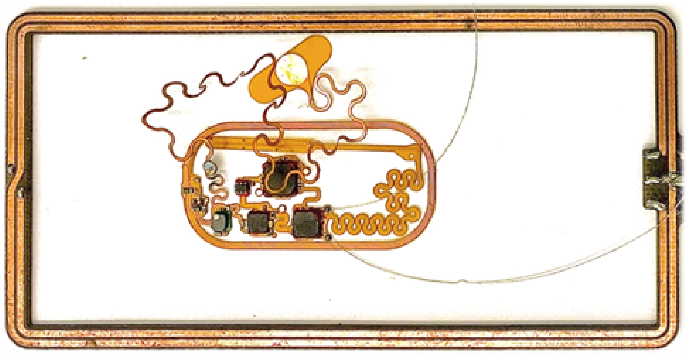

Supplementary Fig. 36. Implant inside a wearable coil integrated with an NFC reader IC.

Publication	Implanted Duration	Electrical Construction	Mechanics	Dimensions	Mode of WPT	Channels	Stimulation Capability	Stimulation Range	Compliance Voltage	Freely moving	Available Power
This Work	6 weeks	Off-the-shelf Components	Flexible	40 mm x 20mm x 1 mm	MRC	8	Biphasic Current Programmable	4.7 mA	± 20V	Yes	120 mW
[35]	Acute	Off-the-shelf Components	Rigid	10.0mm x 8.5 mm x 2.4mm	High Frequency volume conductive	1	Biphasic Constant Current	2mA-4mA	2500 mV	No	5 mW
[36]	Not Available	Off-the-shelf Components	Rigid	11 mm diameter x 5 mm thickness	Ultrasound	1	Voltage Programmable	6V at 10k ohm load	22.16V	No	5.98 mW
[23]	Not Available	ASIC	Rigid	3 mm x 2.15 mm x 14.8 mm	MagnetoElectric	1	Biphasic Voltage Programmable	3.3 V at open load	3300 mV	No	4 mW
[24]	Not Available	ASIC	Rigid	3 mm x 2 mm x 1 mm	Ultrasound	1	monophasic	0.4 mA	3300mV	No	65 µA
[37]	Not Available	Off-the-shelf Components	Rigid	0.5 mm x 0.5 mm x 2.3 mm	Inductive Coupling	1	monophasic	25 µA at 10k ohm load	250 mV	No	5 mW
[38]	Not Available	ASIC	Rigid	2.5 mm x 2.3 mm x 23 mm	MRC	1	Biphasic Current Controlled	0.7 mA	3300 mV	No	3.1 mW
[39]	40 days	ASIC	Rigid	25.3 mm x 9.3 mm x 1.9 mm	Inductive	8	Biphasic Voltage Programmable	100 µA at 2k ohm load	10.5 V	No	Not Available
[40]	4 days	ASIC	Rigid	30 mm x 15 mm x 5 mm	Inductive	36	Biphasic Current	525 µA at 1k ohm	2 V	Yes	43 mW
[41]	2-3 weeks	ASIC	Flexible	12 mm diameter x 0.2 mm	MRC	1	Voltage	N/A	500 mV	No	N/A

Supplementary Table 1. List of wireless battery-free systems for functional electrical stimulation.

Name	Implant Date	Implanted Duration (Days)	Max Current (mA)	Electrode Type	SCI	Failure Mode
Spinal 9	7/2/21	51	1.8 mA	Spinal	No	Amplitude nonprogrammable
Spinal 10	8/2/21	39	1.8 mA	Spinal	No	Communication loss
Spinal 11	8/19/21	7	1.8 mA	Spinal	No	Stimulation during communication (ASK)
Spinal 12	9/2/21	NA	1.8 mA	Spinal	No	In-vitro test failure
Series 61	9/16/21	NA	1.8 mA	Spinal / Muscle	Yes	Surgery failure
Series 62	9/20/21	10	1.8 mA	Spinal / Muscle	Yes	Animal did not survive (SCI complication)
Series 71	10/3/21	NA	1.8 mA	Spinal / Muscle	No	In-vitro test failure
Series 72	10/15/21	NA	1.8 mA	Spinal / Muscle	No	Surgery failure
Series 73	10/27/21	22	1.8 mA	Spinal / Muscle	No	Communication loss
Series 8	11/2/21	16	1.8 mA	Spinal / Muscle	No	Communication loss
Series 9	11/10/21	30	1.8 mA	Spinal / Muscle	No	Communication loss
Series 101	11/23/21	10	1.8 mA	Spinal / Muscle	No	Stimulation during communication (ASK)
Series 102	12/1/21	NA	1.8 mA	Spinal / Muscle	No	In-vitro test failure
Series 11	12/15/21	7	1.8 mA	Spinal / Muscle	No	Stimulation during communication (ASK)
SS 141	1/15/22	37	4.7 mA	Spinal / Muscle	No	Not programmable
SS 142	1/27/22	25	4.7 mA	Spinal / Muscle	No	Communication loss
SS 151	2/9/22	47	4.7 mA	Spinal / Muscle	No	Communication loss
SS 152	3/16/22	30	4.7 mA	Spinal / Muscle	No	Communication loss
SS 16	4/11/22	45	4.7 mA	Spinal / Muscle	No	Communication loss
SS171	5/1/22	50	4.7 mA	Spinal / Muscle	Yes	Communication loss
SS172	5/14/2022	37	4.7 mA	Spinal / Muscle	Yes	Communication loss

Supplementary Table 2. A record of implanted devices, duration of functionality, types, SCI model and failure mode.
