# Supplementary material for: Fully implanted battery-free high power platform for chronic spinal and muscular functional electrical stimulation
Source: Nat Commun. 2023 Nov 30;14:7887. doi: 10.1038/s41467-023-43669-2 (PMC10689769; doi:10.1038/s41467-023-43669-2)
Supplement: Supplementary file 3 — Reporting Summary [file 41467_2023_43669_MOESM3_ESM.pdf]

nature portfolio

Corresponding author(s): Philipp Gutruf

Last updated by author(s): Nov 15, 2023

Reporting Summary

Nature Portfolio wishes to improve the reproducibility of the work that we publish. This form provides structure for consistency and transparency in reporting. For further information on Nature Portfolio policies, see our Editorial Policies and the Editorial Policy Checklist.

Statistics

For all statistical analyses, confirm that the following items are present in the figure legend, table legend, main text, or Methods section.

n/a	Confirmed	
☐	☒	The exact sample size (*n*) for each experimental group/condition, given as a discrete number and unit of measurement
☐	☒	A statement on whether measurements were taken from distinct samples or whether the same sample was measured repeatedly
☐	☒	The statistical test(s) used AND whether they are one- or two-sided<br>*Only common tests should be described solely by name; describe more complex techniques in the Methods section.*
☒	☐	A description of all covariates tested
☐	☒	A description of any assumptions or corrections, such as tests of normality and adjustment for multiple comparisons
☐	☒	A full description of the statistical parameters including central tendency (e.g. means) or other basic estimates (e.g. regression coefficient) AND variation (e.g. standard deviation) or associated estimates of uncertainty (e.g. confidence intervals)
☒	☐	For null hypothesis testing, the test statistic (e.g. F, t, r) with confidence intervals, effect sizes, degrees of freedom and P value noted<br>*Give P values as exact values whenever suitable.*
☒	☐	For Bayesian analysis, information on the choice of priors and Markov chain Monte Carlo settings
☒	☐	For hierarchical and complex designs, identification of the appropriate level for tests and full reporting of outcomes
☒	☐	Estimates of effect sizes (e.g. Cohen's *d*, Pearson's *r*), indicating how they were calculated

Our web collection on statistics for biologists contains articles on many of the points above.

Software and code

Policy information about availability of computer code

Data collection	Data collection is stated in detail in the methods section. Software used - AutoCAD 2021, Ansys Electronic Desktop (AED) 2021 R1, ImageJ 1.53t, Nucline v2.01, DeepLabCut 2.2.0
Data analysis	All data analysis software is identified in the methods section. Software used - Ansys Electronic Desktop (AED) 2021 R1, Ansys Simplorer 11.0, Ansys 2019 R2 Static Structural, Amira 2020.2

For manuscripts utilizing custom algorithms or software that are central to the research but not yet described in published literature, software must be made available to editors and reviewers. We strongly encourage code deposition in a community repository (e.g. GitHub). See the Nature Portfolio guidelines for submitting code & software for further information.

Data

Policy information about availability of data

All manuscripts must include a data availability statement. This statement should provide the following information, where applicable:

- Accession codes, unique identifiers, or web links for publicly available datasets
- A description of any restrictions on data availability
- For clinical datasets or third party data, please ensure that the statement adheres to our policy

The source data generated in the study has been deposited in and available at the University of Arizona Data Repository and can be accessed from https://doi.org/10.25422/azu.data.24329134.v1. All the experimental data generated in this study are provided in the Supplementary Information/Source Data file. Additional data not covered in this document can be made available from the authors upon request.

Research involving human participants, their data, or biological material

Policy information about studies with human participants or human data. See also policy information about sex, gender (identity/presentation), and sexual orientation and race, ethnicity and racism.

Reporting on sex and gender	N/A
Reporting on race, ethnicity, or other socially relevant groupings	N/A
Population characteristics	N/A
Recruitment	N/A
Ethics oversight	N/A

Note that full information on the approval of the study protocol must also be provided in the manuscript.

Field-specific reporting

Please select the one below that is the best fit for your research. If you are not sure, read the appropriate sections before making your selection.

- [x] Life sciences
- [ ] Behavioural & social sciences
- [ ] Ecological, evolutionary & environmental sciences

For a reference copy of the document with all sections, see nature.com/documents/nr-reporting-summary-flat.pdf

Life sciences study design

All studies must disclose on these points even when the disclosure is negative.

Sample size	The sample size was not predetermined as the animal experiments of this study are focusing on establishing the functionality of the devices. We thus tested the device stability by chronically and continuously testing the device functions implanted in the animals for multiple weeks.
Data exclusions	No data were excluded from the analyses.
Replication	We use standardized procedures for implant surgery and data collection. All the analyses are also done by the same codes and algorithms.
Randomization	This study is to test the functionality of a newly developed device. The randomization is not applicable in this study.
Blinding	As this study focuses on testing the functionality, blinding is not relevant.

Reporting for specific materials, systems and methods

We require information from authors about some types of materials, experimental systems and methods used in many studies. Here, indicate whether each material, system or method listed is relevant to your study. If you are not sure if a list item applies to your research, read the appropriate section before selecting a response.

Materials & experimental systems

n/a	Involved in the study
☒	☐ Antibodies
☒	☐ Eukaryotic cell lines
☒	☐ Palaeontology and archaeology
☐	☒ Animals and other organisms
☒	☐ Clinical data
☒	☐ Dual use research of concern
☒	☐ Plants

Methods

n/a	Involved in the study
☒	☐ ChIP-seq
☒	☐ Flow cytometry
☒	☐ MRI-based neuroimaging

Animals and other research organisms

Policy information about studies involving animals; ARRIVE guidelines recommended for reporting animal research, and Sex and Gender in Research

Laboratory animals	Detailed information on animals is found in the methods section. Animals used are Female Sprague-Dawley rats (250-350 g).
Wild animals	Animals can found in the methods section. No wild animals are used
Reporting on sex	Sex is reported.
Field-collected samples	No field collected sample was used in the study.
Ethics oversight	Institutional Animal Care and Use Committee of Northwestern University (protocol number: IS00011700).

Note that full information on the approval of the study protocol must also be provided in the manuscript.
